# Predictive Modeling of Tensile Strength in Aluminum Alloys via Machine Learning

**DOI:** 10.3390/ma16227236

**Published:** 2023-11-20

**Authors:** Keya Fu, Dexin Zhu, Yuqi Zhang, Cheng Zhang, Xiaodong Wang, Changji Wang, Tao Jiang, Feng Mao, Cheng Zhang, Xiaobo Meng, Hua Yu

**Affiliations:** 1 School of Electrical & Information Engineering, Beihang University, No. 37, Xueyuan Road, Beijing 100191, China; sniper_fox007@163.com; 2 Beijing Advanced Innovation Center for Materials Genome Engineering, Innovation Research Institute for Carbon Neutrality, University of Science and Technology Beijing, No. 30, Xueyuan Road, Beijing 100083, China; 18939656652@163.com; 3 State Key Laboratory for Advanced Metals and Materials, University of Science and Technology Beijing, No. 30, Xueyuan Road, Beijing 100083, China; zhang18438698876@163.com (Y.Z.);; 4 National Joint Engineering Research Center for Abrasion Control and Molding of Metal Materials, Henan University of Science and Technology, Luoyang 471003, China; 5 Longmen Laboratory, Luoyang 471003, China; 6 School of Materials Science and Engineering, Henan University of Science and Technology, Luoyang 471003, China

**Keywords:** aluminum alloy, machine learning, tensile strength, polynomial regression

## Abstract

Aluminum alloys are widely used due to their exceptional properties, but the systematic relationship between their grain size and their tensile strength has not been thoroughly explored in the literature. This study aims to fill this gap by compiling a comprehensive dataset and utilizing machine learning models that consider both the alloy composition and the grain size. A pivotal enhancement to this study was the integration of hardness as a feature variable, providing a more robust predictor of the tensile strength. The refined models demonstrated a marked improvement in predictive performance, with XGBoost exhibiting an *R^2^* value of 0.914. Polynomial regression was also applied to derive a mathematical relationship between the tensile strength, alloy composition, and grain size, contributing to a more profound comprehension of these interdependencies. The improved methodology and analytical techniques, validated by the models’ enhanced accuracy, are not only relevant to aluminum alloys, but also hold promise for application to other material systems, potentially revolutionizing the prediction of material properties.

## 1. Introduction

Aluminum alloys possess commendable mechanical properties, such as a high strength-to-weight ratio, excellent corrosion resistance, an appealing appearance, good recyclability, ease of manufacture, and non-magnetism. In recent years, aluminum alloys have found extensive applications in large-span structures, marine and offshore structures, movable lightweight structures, and prefabricated systems [1,2]. Over the past few decades, numerous studies have been conducted on the material properties of aluminum alloys and their structural performance at environmental temperatures [3,4,5,6,7,8,9]. All of these studies have improved the properties of aluminum alloys by adding elements, biphasic nucleation, and new preparation processes. However, it should be noted that these improvement methods are closely related to the grain size. About 70 years ago, it was discovered that there is a linear relationship between flow stress and the square root of the grain size, resulting in the recognized Hall-Petch grain refinement and strengthening effect [10]. Since then, researchers have also focused on the crystal size and properties of aluminum alloys. For example, the relationship between the grain size and properties such as the flow stress [11,12], mechanical properties [13], thermal cracking [14], and deformation temperature [15] have been examined. Although grain refinement can enhance the performance of aluminum alloys, abnormal grain growth can lead to structural defects, severely affecting the mechanical properties of the alloys [16,17,18]. With the development of computers and information technology, mathematical models describing the structure and properties are the basis for the accurate prediction and control of the structure and properties of aluminum alloys in the production process, and thus, they have become a focus of attention [19,20,21,22]. However, these current predictive models are mainly obtained by regressing experimental data on specific cases or by physical/mathematical reasoning under certain assumptions.

In recent years, machine learning has transcended its theoretical boundaries, demonstrating practical applications across diverse fields, particularly in materials science [23,24,25,26,27]. These techniques, distinguished by their data-intensive approach, enable nuanced understanding and prediction by identifying complex patterns and relationships within extensive datasets. For instance, researchers have applied machine learning algorithms to predict hardness, yield/tensile strength, and elastic constants with a considerable accuracy [28,29,30]. These studies exemplify the potential of machine learning to facilitate a deeper understanding of material behavior, optimize material performance, and even guide the discovery of new materials. Moreover, machine learning’s predictive capabilities are not confined to homogeneous or isotropic materials, but extend to anisotropic materials, composites, and intricate material systems. Such versatility underscores the adaptability and broad applicability of machine learning in contemporary materials science research. However, despite these advancements, there is a noticeable gap in the application of machine learning to the realm of aluminum alloys, particularly concerning the prediction of tensile strength based on compositional variables and the grain size. This research endeavors to bridge this gap, contributing to the burgeoning field of materials informatics and offering new insights for the production and application of aluminum alloys.

This study explored the intricate relationships between the tensile strength of aluminum alloys, the alloy composition, and the grain size. It utilized a diverse range of machine learning algorithms to model and predict the tensile strength of these aluminum alloys. Through different feature selection methods and model evaluations, the most influential features were determined, thereby more effectively revealing the correlation between elemental feature variables and the grain size with the tensile strength. Furthermore, a mathematical expression was derived to quantitatively assess the correlation between the tensile strength and the aluminum alloy composition and grain size, employing a polynomial regression analysis.

## 2. Data Preparation and Analysis

### 2.1. Data Collection and Preprocessing

Data collection constitutes the foundation of materials informatics research. To investigate the correlation between the tensile strength of aluminum alloys and their alloy composition and grain size, 84 experimental data points were gathered from the literature [31,32,33,34,35,36,37,38,39,40,41,42,43,44,45,46,47,48,49,50,51,52,53,54,55]. The vast majority of the data in the dataset (90%) were cast aluminum alloys, with the remainder being wrought aluminum alloys. Following the data collection, a series of data preprocessing operations were conducted to ensure data quality and consistency. This preprocessing stage was pivotal in ensuring the accuracy and reliability of the analysis, encompassing steps such as data cleaning and outlier handling. During the data-cleaning phase, incomplete or inaccurate data points that could potentially introduce interference were eliminated. Simultaneously, outlier handling was performed to exclude alloy element combinations that appeared infrequently in the entire dataset of aluminum alloy tensile strength, thereby enhancing the representativeness and credibility of the dataset. Notably, data points with incomplete alloy component information and alloy element combinations that only occurred once were removed during the data preprocessing process, to augment the completeness and significance of the dataset.

Consequently, after data preprocessing, a dataset comprising 67 data points of aluminum alloy tensile strength was obtained. The dataset encompassed information on the alloy composition, the grain size, and the corresponding tensile strength of the aluminum alloy samples. It is worth mentioning that this dataset encompassed 19 chemical elements, with each sample’s data including 19 chemical element variables, 1 grain size variable, and 1 target performance variable of tensile strength. These features provided a pool of information for a subsequent analysis and modeling. In Table 1, the minimum, maximum, and mean values of each feature variable are listed to provide a comprehensive understanding of the dataset’s distribution features.

### 2.2. Data Analysis

**Figure 1:**
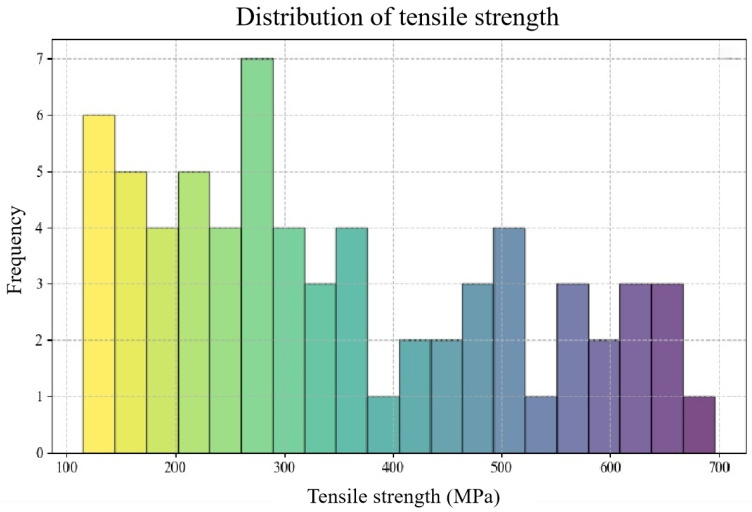
Histogram of frequency distribution of tensile strength of aluminum alloys.

Data analysis serves as a crucial step in gaining a profound understanding of the relationship between the tensile strength of aluminum alloys and various feature variables. We adopted two methods, data visualization and a regression analysis from a statistical analysis, to comprehensively reveal these relationships. Data visualization played a pivotal role in this study, as it facilitated an intuitive understanding of the data features and their interrelations through a graphical representation. Conversely, a regression analysis was employed to construct machine learning models, thereby providing a deeper insight into the connections between the tensile strength of aluminum alloys and each feature variable. In this section, a frequency distribution histogram of the tensile strength of aluminum alloys is plotted, as shown in Figure 1. Through this frequency distribution histogram, the distribution of the tensile strength data can be comprehensively understood, aiding in the comprehension of the data features and statistical properties.

**Figure 2:**
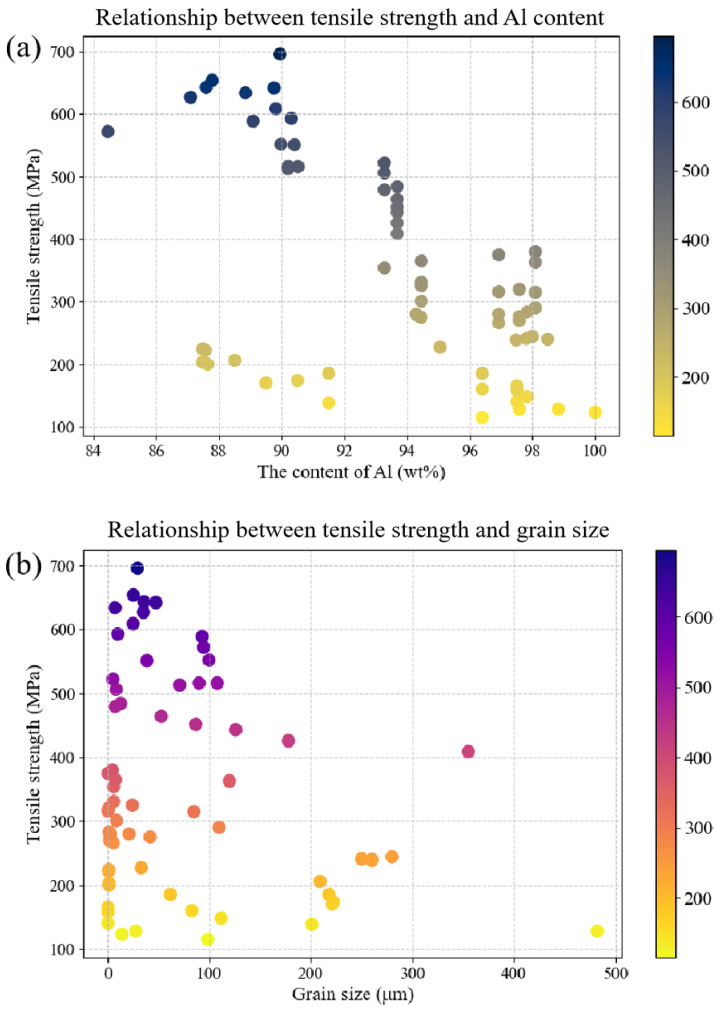
Scatter plots depicting the relationship between aluminum content, grain size, and tensile strength of aluminum alloys: (**a**) shows the correlation between aluminum content and tensile strength, and (**b**) illustrates the relationship between grain size and tensile strength.

Figure 2 comprises two scatter plots that provide valuable insights into the relationship between various parameters and the tensile strength of aluminum alloys. The scatter plots in Figure 2 provide clear evidence of the impact of the aluminum content and grain size on the tensile strength of aluminum alloys. Specifically, the data reveal a negative correlation between the aluminum content and the tensile strength, with an increase in the aluminum content leading to a decrease in the tensile strength. Additionally, the scatter plot for grain size and tensile strength showed a similar trend, with larger grain sizes resulting in a lower tensile strength. These findings offer valuable insights into the mechanical properties of aluminum alloys and highlight the importance of carefully controlling these factors to ensure an optimal performance in practical applications.

## 3. Machine Learning Algorithms and Evaluation Methods

### 3.1. Machine Learning Algorithm and Parameter Settings

This study employed different machine learning regression algorithms, all of which originated from the open-source algorithm package in scikit-learn [56]. Here is a brief introduction to each algorithm: Linear regression is a classic linear model, suitable for modeling simple linear relationships. Random forest is applicable to complex non-linear problems, with the number and depth of trees being significant parameters. GBDT (gradient-boosting decision trees) is an ensemble of learning algorithms that improves the prediction performance by iteratively training decision tree models. GBDT optimizes the loss function using gradient descent to gradually reduce the prediction error and improve the overall model performance. The performance of the K-nearest neighbors algorithm is greatly influenced by the choice of K. Neural networks simulate connections between neurons in the human brain, with the key parameters including the number of network layers, the number of neurons, and the learning rate. XGBoost is a gradient-boosting algorithm, performing exceptionally well in regression and classification problems, with the important parameters including the learning rate and the depth of trees. LightGBM efficiently handles large-scale datasets and is suitable for high-performance machine learning tasks, with the key parameters including the learning rate, the number of trees, and their depth. Unless specifically stated, the default parameter settings for these algorithms were derived from scikit-learn and were carefully selected in the algorithm implementation, typically suitable for various types of problems. These algorithms were employed to capture the relationship between feature variables (alloy compositions and grain size) and target variables (tensile strength). The diversity and flexibility of the algorithms contributed to a comprehensive exploration and understanding of the correlation between the tensile strength of aluminum alloys and the feature variables, thereby providing a powerful tool for research in the field of materials science.

### 3.2. Model Evaluation Methods and Evaluation Indicators

Ten-fold cross-validation is a commonly used method for assessing the performance of machine learning models, and is particularly suitable for small sample datasets. It divides the original dataset into ten equal subsets, sequentially using each subset as the test set and the remaining nine subsets combined as the training set. The model is trained and evaluated on each test set, yielding ten independent performance evaluation results. Ultimately, the average of these ten evaluation results is computed to obtain the final performance evaluation metric. Ten-fold cross-validation has a distinct advantage when dealing with small sample datasets: by randomly dividing the training and test sets multiple times, it reduces the variance of the evaluation results, thereby enhancing the stability of the evaluation. Every sample has the opportunity to serve as the test data, and the performance of each model is thoroughly assessed, thus providing a more comprehensive understanding of the model’s performance. For small sample datasets, ten-fold cross-validation helps prevent overfitting on the training set and improves the evaluation of the model’s generalization ability for unseen data.

In this paper, evaluation metrics, including *R^2^* (coefficient of determination) and the *RMSE* (root mean squared error), were utilized to measure the effectiveness of each algorithm in modeling the relationship between the tensile strength of aluminum alloys and the feature variables. *R^2^*, also known as the coefficient of determination, is an important metric for measuring the degree of fit of a regression model to observed data. It represents the proportion of the variance in the target variable that is predictable from the feature variables. The formula for calculating *R^2^* is as follows:

(1) $$R^{2}=1-\frac{\sum_{i=1}^{n}{\left(y_{i}-{\hat{y}}_{i}\right)}^{2}}{\sum_{i=1}^{n}{\left(y_{i}-\bar{y}\right)}^{2}}$$

In the formula, $y_{i}$ and ${\hat{y}}_{i}$ are the observed and corresponding predicted values, respectively, while $\bar{y}$ and $\bar{\hat{y}}\;$are the mean values of $y_{i}$ and $\hat{y}$, respectively. The range of *R^2^* lies between 0 and 1. When *R^2^* equals 1, it indicates a perfect fit of the model to the data; conversely, when *R^2^* equals 0, the model fails to explain any variance in the target variable, thereby representing the worst fit.

The *RMSE/MSE/MAE* is utilized to measure the prediction error of the regression model, reflecting the average discrepancy between the model’s predictions and the actual observations. A smaller value indicates a higher model prediction accuracy, i.e., a smaller difference between the predicted and actual values. The formulas for calculating the *RMSE/MSE/MAE* are as follows:

(2) $$RMSE=\sqrt{\sum_{i=1}^{n}\frac{1}{n}{\left({\hat{y}}_{i}-y_{i}\right)}^{2}}$$

(3) $$MSE=\frac{1}{n}\sum_{i=1}^{n}\left({\hat{y}}_{i}-y_{i}\right)$$

(4) $$MAE=\frac{1}{n}\left|{\hat{y}}_{i}-y_{i}\right|$$

In these formulas, $y_{i}\;$represents the observed values, ${\hat{y}}_{i}$ represents the model’s predicted values, and n denotes the number of samples. The range of the *RMSE/MSE/MAE* typically aligns with the unit of the target variable. A smaller *RMSE/MSE/MAE* value suggests a smaller prediction error of the model, i.e., the model is closer to the actual data. All of these evaluation metrics, *R^2^* and the *RMSE/MSE/MAE*, played a pivotal role in the regression analysis. They provided an important basis for objectively assessing the model performance.

## 4. Results and Discussion

### 4.1. Modeling Based on Aluminum Alloy Composition and Grain Size

In this study, we initially embarked on machine learning modeling based on the composition of aluminum alloys (comprising 18 elements), the grain size, and the target property of tensile strength. Distinct machine learning algorithms were employed, namely linear regression (LR), random forest (RF), k-nearest neighbors (KNN), extreme gradient boosting (XGBoost), the light gradient-boosting machine (LightGBM), and an artificial neural network (ANN). These models were constructed to predict the tensile strength of aluminum alloys based on their composition and grain size. The modeling process underwent a rigorous evaluation, and the ten-fold cross-validation method was adopted, thereby ensuring the reliability of the model’s performance.

Table 2 presents the performance evaluation results of each model, encompassing the different metrics. The linear regression model yielded an *R^2^* value of 0.55 and an *RMSE* of 171.51 MPa. Although its performance was relatively low, it provided a benchmark for subsequent model comparisons. The random forest model exhibited an exceptional performance with an *R^2^* value of 0.86 and an *RMSE* of merely 62.42 MPa, indicating its efficacy in capturing the intricate relationship between the tensile strength of the aluminum alloys and the grain size. The KNN model, however, achieved an *R^2^* value of 0.39 and an *RMSE* of 129.04 MPa, suggesting that it might be less suitable for problems with a plethora of features. KNN, in its essence, predicts the label of a new data point by considering the labels of its “nearest” neighbors within the feature space. While renowned for its simplicity and effectiveness in scenarios with fewer dimensions, KNN’s efficacy diminishes in the face of high-dimensional data, a phenomenon often termed the “curse of dimensionality.” Furthermore, our dataset, rich in features, included variables of varying relevance to the tensile strength prediction, introducing noise into the distance calculations pivotal to KNN and inadvertently leading to the model recognizing incorrect neighbors. Compounding these challenges was the issue of data sparsity: the exponential increase in feature space volume inherent with higher dimensions results in sparser data, a situation ill-suited for KNN, especially given our dataset’s modest size of 67 data points. The XGBoost model demonstrated an impressive ability to address the relationship between the aluminum alloy tensile strength and the grain size, with an *R^2^* value of 0.88 and an *RMSE* of 57.72 MPa. The LightGBM model’s performance was closely aligned with XGBoost, registering an *R^2^* value of 0.77 and an *RMSE* of 80.06 MPa. The ANN model, although slightly inferior to random forest, XGBoost, and LightGBM, still produced an acceptable *R^2^* value of 0.73 and an *RMSE* of 85.69 MPa. These evaluation results unequivocally highlight that sophisticated models such as random forest, XGBoost, and LightGBM excel in modeling the relationship between the aluminum alloy tensile strength and the grain size, possibly due to their capacity to capture more complex non-linear relationships. Conversely, linear regression and KNN exhibited a lower accuracy on this issue.

### 4.2. Feature Selection and Modeling

Feature selection stands as a pivotal step in machine learning and data analysis, focusing on the effective reduction in data dimensions without compromising essential information. In the context of studying the tensile strength of aluminum alloys, feature selection becomes paramount. Considering the numerous and intricate components of aluminum alloys, it is crucial to pinpoint the key elements that genuinely impact the tensile strength. Traditional statistical methods, such as a correlation analysis and a variance analysis, are no longer sufficient for this purpose. Therefore, we also turned to advanced machine learning techniques. In this research, the random forest model was employed, which constructs multiple decision trees and amalgamates their predictive outcomes, thereby enhancing the prediction accuracy. The random forest model assigns importance scores to each feature, aiding in the identification of the features with the most significant impact on the target variable. However, relying solely on the feature importance scores from random forest might be inadequate. To achieve a more comprehensive and equitable feature evaluation, we introduced an advanced feature interpretation technique based on Shapley values—SHAP (Shapley additive explanation) values [57,58]. In this study, we incorporated an in-depth explanation and application of SHAP (Shapley additive explanation) values to enhance the transparency and interpretability of our machine learning models. SHAP values, which represent a prominent method in the domain of machine learning interpretability, offer significant advantages over traditional feature importance metrics by providing a detailed contribution of each feature for individual predictions, rather than just an aggregate impact. The concept of SHAP values is rooted in cooperative game theory, wherein the Shapley values allocate fair “payouts” to players in a coalition based on their contribution. In the context of our machine learning model, SHAP values assign each feature a value indicative of its contribution to a specific prediction, thus offering a granular understanding of the model’s decision-making process. In our methodology, we applied SHAP values to our random forest model, enabling us to delve into the intricate relationship between our predictors (such as aluminum content, grain size, etc.) and the target outcome (tensile strength). This method illuminated critical insights into both global and local feature importance, thereby significantly enriching our analysis and conclusions. In contrast to the feature importance scores obtained from random forest, SHAP values provide a more comprehensive and fair assessment of features. By synthesizing the insights from the random forest model and SHAP values, we discerned the influence of different feature variables on the tensile strength.

**Figure 3:**
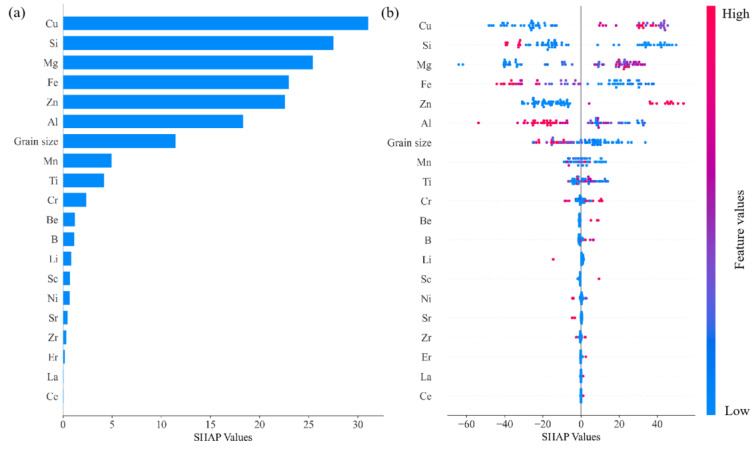
Analysis results of SHAP values; (**a**) importance ranking of element features for tensile strength and (**b**) summary plot of SHAP values.

The magnitude of the SHAP value reflects the importance of a specific feature. Figure 3a provides a summary of the key factors influencing the tensile strength, with Cu, Si, Mg, Fe, and Zn identified as the top five features. In Figure 3b, the SHAP values quantify the individual contributions of these features to the target variable. Positive SHAP values indicate a positive effect, while negative values indicate a negative correlation. Figure 3b, the SHAP summary plot, visually represents the SHAP values for each feature across all samples. Based on Figure 3b, it can be inferred that there is an inverse relationship between the Al content and the grain size with respect to the tensile strength.

Subsequently, by focusing on these five elemental feature variables (Cu, Si, Mg, Fe, and Zn), the optimal subset method was employed for further feature selection, aiming to ascertain which elemental combinations play a pivotal role in establishing the optimal tensile strength prediction model. The optimal subset method is a feature selection technique that determines the best predictive model combination by considering various feature variable combinations. In this instance, the top five elemental feature variables (Cu, Si, Mg, Fe, and Zn) were selected as the candidate feature set.

**Figure 4:**
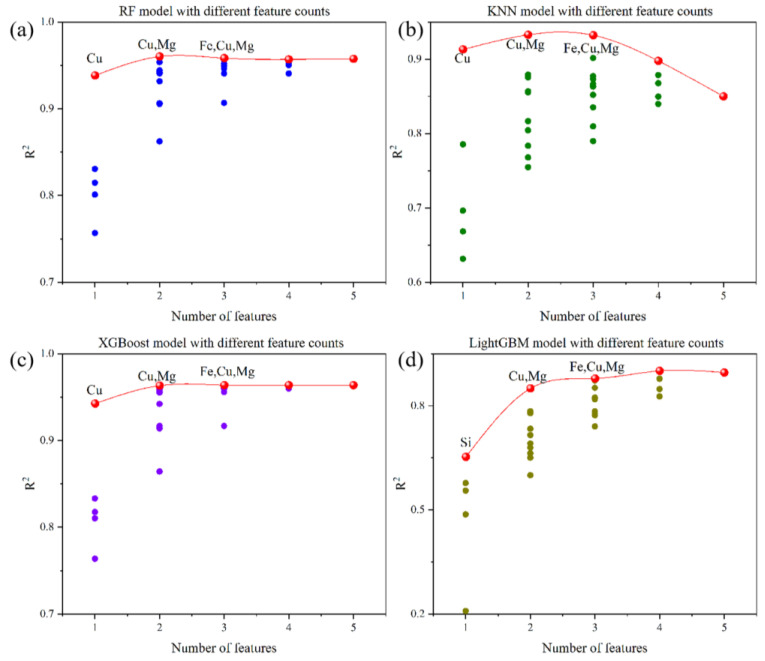
Importance ranking of element features for tensile strength of Al alloys: (**a**) RF, (**b**) KNN, (**c**) XGBoost, and (**d**) LightGBM, respectively.

By utilizing the optimal subset method [59,60], four distinct machine learning algorithms were explored, namely random forest, k-nearest neighbors, LightGBM, and XGBoost. For each algorithm, the best feature combination was identified by comparing the performance of various feature sets. From the importance ranking of element features in Figure 4a–d, it can be seen that in different ML models, the model generally has the highest *R^2^* when the number of element features is 3. After a thorough analysis and comparison, the optimal variable combination was determined, encompassing the elements Fe, Cu, and Mg. This aided in further simplifying the model, reducing the feature dimensions, enhancing the model interpretability, and, consequently, decreasing the model complexity. By selecting the optimal feature combination, we could more effectively capture the relationship between elemental feature variables and the tensile strength, while also reducing computational costs. This step allowed for a more precise determination of which elements significantly influence the tensile strength and their interplay. This facilitated the construction of simpler models that still possessed high predictive capabilities, offering deeper insights for understanding and optimizing material properties. Through feature selection, attention can be more effectively directed towards the elemental feature variables that have a pivotal impact on the tensile strength prediction, further optimizing the material design and refinement processes.

The three elemental feature variables derived from the feature selection, along with the grain size, were used as the inputs for different machine learning models for further modeling. Automated machine learning, commonly known as AutoML, streamlined the process of selecting the appropriate algorithms and tuning the hyperparameters to achieve an optimal model performance. Particularly, the flaml package is a Python library that automates this process and offers a fast and efficient framework for hyperparameter optimization. After our initial modeling with machine learning algorithms, we further employed AutoML, specifically leveraging the flaml package, for hyperparameter tuning. This additional step of optimization enhanced the precision of our models, making them more robust and accurate in predicting the tensile strength of aluminum alloys. Table 3 provides the detailed results of the top four ML models using different evaluation metrics.

**Figure 5:**
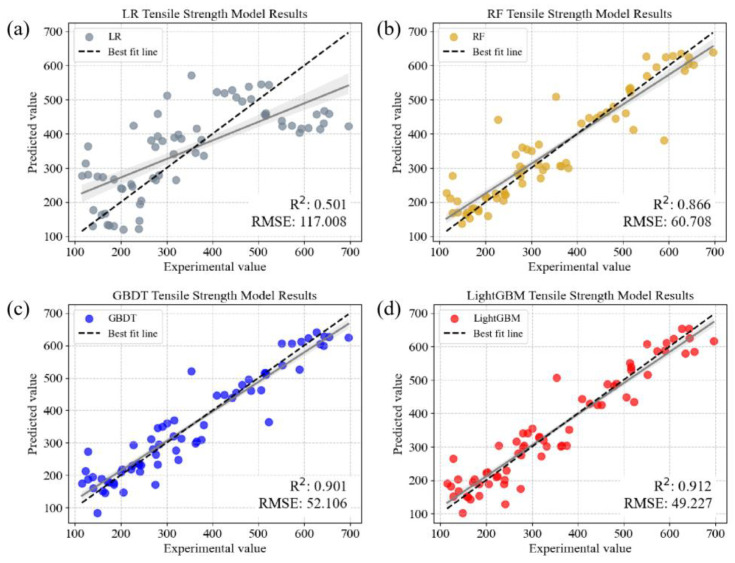
Scatter plot of comparison of experimental values with predicted values of different models after feature selection: (**a**) LR, (**b**) RF, (**c**) GBDT, and (**d**) LightGBM, respectively. (all models were evaluated with 10-fold cross-validation and the grey line represents the fit curve with an 80% confidence interval).

To comprehensively understand the performance of the model, a scatter plot was created for the top four models based on *R^2^*. The plot included the predicted values and experimental values, along with a 45° diagonal line and confidence intervals. This was implemented to observe the relationship and deviations between the predicted results and the actual observations. Ideally, the points on the scatter plot should cluster around the 45° diagonal line, indicating a perfect match between the model predictions and the experimental values. Therefore, a denser distribution of points near the diagonal line signifies a high congruence between the model predictions and the actual observations. A further performance analysis clearly revealed that sophisticated models such as RF, GBDT, and LightGBM excel in modeling the relationship between the aluminum alloy tensile strength and the grain size, as shown in Figure 5b–d. Their predicted points were more concentrated around the 45° diagonal line, showcasing a superior predictive accuracy. Conversely, models such as linear regression exhibited an underperformance in Figure 5a, as their predicted points were more dispersed and displayed less alignment with the diagonal line. The results from Figure 5 indicate that, compared to the initial nineteen elemental variables, the performance of the three selected elemental variables was comparable, underscoring the efficacy of the proposed feature selection method. This selection not only filtered out features with lower associations to the tensile strength, but also further reduced the risk of overfitting and training costs, enhancing the model interpretability.

Despite diligent feature selection and hyperparameter optimization, the outcomes of our models did not meet the desired level of satisfaction. In an endeavor to enhance the predictive capability of our models, we revisited the dataset and extracted hardness values for the corresponding aluminum alloys from the original literature. Hardness, as a mechanical property, was anticipated to provide a substantial correlation with the tensile strength, thereby potentially elevating the predictive accuracy of the models. It is noteworthy, however, that not all sources provided measurements for hardness; thus, we were able to augment our dataset with hardness values for only 38 data points. Nevertheless, the incorporation of this mechanical property into our analysis yielded gratifying results. The inclusion of hardness as an additional feature variable in the predictive modeling of aluminum alloy tensile strength led to noteworthy improvements in the performance of our machine learning models, as shown in Table 4.

**Figure 6:**
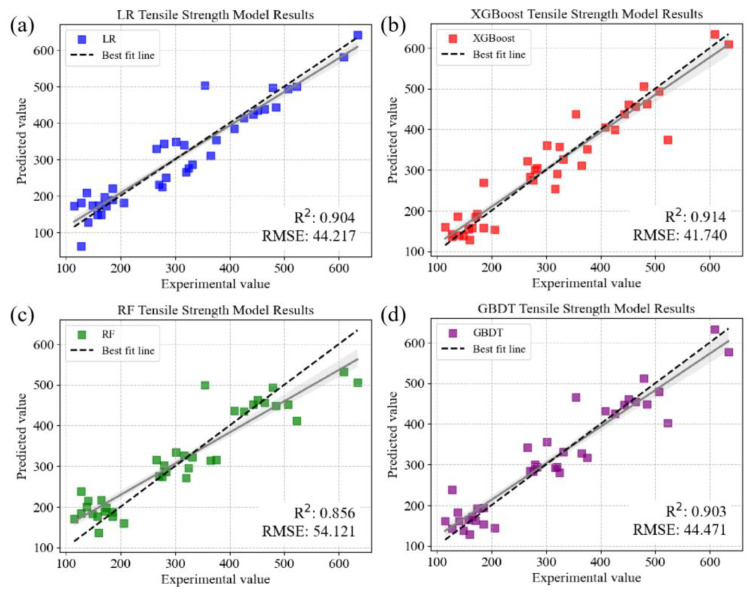
Scatter plot of comparison of experimental values with predicted values of different models using screened composition, grain size, and hardness: (**a**) LR, (**b**) XGBoost, (**c**) RF, and (**d**) GBDT, respectively. (all models were evaluated with 10-fold cross-validation and the grey line represents the fit curve with an 80% confidence interval).

The LR model, in particular, demonstrated a significant leap in predictive accuracy, with its *R^2^* value ascending from 0.501 to 0.904 and the *RMSE* sharply declining from 117.008 MPa to 44.217 MPa. This substantial enhancement in the LR indicated that the relationship between the predictive features and the tensile strength became more linear with the integration of hardness, thereby refining the model’s interpretative and predictive capacity. XGBoost also showed positive gains, with its *R^2^* increasing marginally from 0.884 to 0.914 and its *RMSE* decreasing to 41.740 MPa, suggesting that the inclusion of hardness fine-tuned the model’s ability to capture the complex interplay between the features and the tensile strength. Conversely, the RF model exhibited a slight decrease in its *R^2^* value from 0.891 to 0.854, yet sustained a steady *RMSE* of 54.447 MPa, indicating a robustness in its predictive precision, despite the integration of the additional feature. The slight dip in *R^2^* may be attributed to the RF model’s complexity and the potential redundancy introduced by the correlated features. The subsequent analysis introduced GBDT in lieu of LightGBM, with GBDT continuing the trend of a high performance, as evidenced by an *R^2^* of 0.907 and an *RMSE* of 43.470 MPa. This performance was consistent with the high predictive accuracy previously established by LightGBM, reaffirming the strength of gradient-boosting methodologies in this research domain. The scatter plot in Figure 6 shows the comparison between the predicted values and the experimental values for each of the four models. In summary, these results underscore the pivotal role of feature engineering in materials informatics. By selecting features underpinned by both statistical significance and physical relevance to the property of interest, the precision and reliability of predictive models can be improved. The strategic inclusion of hardness provided an additional layer of insight, empowering our models to unravel the complex dependencies and, thus, predict the tensile strength of aluminum alloys with an enhanced accuracy.

### 4.3. Polynomial Regression and Analysis

In this section, we delve deeply into the pivotal role of a polynomial regression analysis in predicting the tensile strength of aluminum alloys [61]. The objective of this analysis was to elucidate the intricate mathematical relationships between the tensile strength and key elemental variables, as well as the grain size, thereby offering a robust tool for a more profound understanding and optimization of material properties. The choice of a polynomial regression analysis stems from an understanding of the complexity of aluminum alloy material properties, as this method is adept at capturing non-linear relationships, thus providing a more accurate depiction of the influence of the elemental composition on the tensile strength. A salient advantage of this approach lies in its ability to handle intricate relationships, encompassing higher-order polynomial terms, to comprehensively consider the impact of the elemental composition. When constructing the mathematical model, higher-order polynomial terms of the chosen elemental variables were considered, and their relationships with the tensile strength were quantified through regression coefficients. Regarding the polynomial regression model, its inherent characteristic of relying on the entire dataset to generate a single, global equation makes it unsuitable for cross-validation. Therefore, in this case, the entire dataset was utilized to train the polynomial regression model. The mathematical relationship derived from the polynomial regression is represented below:

(5) $$\begin{array}{c} Ts=236.03-180.52\times Fe+151.99\times Cu+24.63\times Mg-0.58\times Gs\\ \;\;\;\;\;\;\;\;\;-140.19\times Fe^{2}+1.24\times Fe\times Gs-41.94\times Cu^{2}+53.41\times Cu\times Mg \end{array}$$

In Equation (5), Ts represents the tensile strength of the aluminum alloy while Fe, Cu, and Mg denote the content of iron, copper, and magnesium elements in the aluminum alloy, respectively, and Gs signifies the grain size. The coefficients preceding each variable are regression coefficients, illustrating the specific impact of each element and the grain size on the tensile strength. According to Equation (5), the following key results can be observed: the tensile strength is inversely proportional to the content of Fe and the grain size, and directly proportional to the content of Cu and Mg. These results are consistent with previous research findings. It is known from this study that, with an increase in the Fe/Si ratio, there is a notable decline in the tensile strength in the rolling direction, transverse direction, and 45° direction, and elongation significantly increases; when the Fe/Si ratio increases to between 3 and 3.4, the decrease in β slows down and stabilizes, and δ experiences a significant reduction. Copper is a crucial alloying element with a certain solid-solution-strengthening effect, and the precipitated CuAl_2_ during aging also has a remarkable aging-strengthening effect. The copper content in aluminum sheets is typically between 2.5% and 5%, and the strengthening effect is optimal when the copper content is between 4% and 6.8%; hence, most high-strength aluminum alloys have a copper content within this range. Magnesium significantly strengthens aluminum; for every 1% increase in magnesium, the tensile strength increases by approximately 34 MPa. By providing the content of the elements Fe, Cu, and Mg in an aluminum alloy and the grain size Gs, the tensile strength of the aluminum alloy can be calculated through Equation (5).

**Figure 7:**
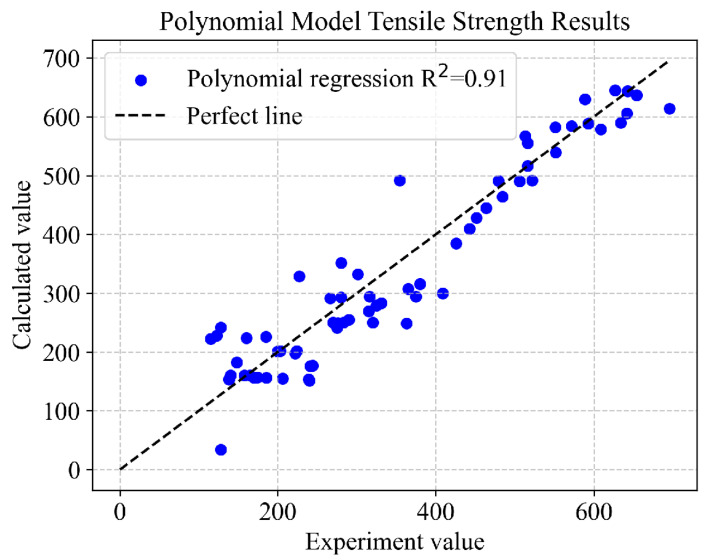
Experimental values versus values calculated from the proposed equation (equation obtained by fitting 67 data points from the entire dataset).

To validate the accuracy of the established polynomial regression model, a comparison was made between the experimental data and the model-predicted values, revealing that the formula possesses an excellent predictive accuracy, achieving an *R^2^* = 0.91, as shown in Figure 7. This substantiates that the polynomial regression analysis is effective and applicable for the design and performance prediction of aluminum alloy materials. Although our dataset was comprehensive, it did not encompass the exhaustive spectrum of variables that could potentially influence the extrapolation of our findings. We are also aware of the inherent assumptions imposed by each machine learning model employed in our study, ranging from the assumption of linearity in our regression models to the nuanced ramifications of hyperparameter selections in more complex models such as random forest and XGBoost. Furthermore, the utilization of SHAP values for feature interpretability, despite its robustness, remains susceptible to the complex uncertainties that accompany the interpretation of intricate, high-dimensional data interactions. An acknowledgment must be made of this study’s specific contextual framework, underscoring that the applicability of the findings could be confined to the distinct types of aluminum alloys and manufacturing conditions examined. The recognition of these limitations underscores the necessity of prudence in extrapolating these conclusions to more expansive contexts.

## 5. Conclusions

In this study, we developed predictive machine learning models that leverage not only the chemical composition, but also the grain size to forecast the tensile strength of aluminum alloys. The models were refined through meticulous feature selection and comprehensive model evaluations. It was discerned that the integration of the grain size with key elemental constituents—namely Fe, Cu, and Mg—imparted a pronounced influence on the tensile strength. The inclusion of hardness as an additional feature notably augmented the models’ predictive ability. Specifically, the XGBoost model exhibited exceptional proficiency in predicting the tensile strength, with an *R^2^* of 0.914. This study substantiates the critical role of feature selection in reducing data dimensionality and streamlining the models, underscoring that the identification and utilization of the most salient features can more adeptly unravel the complex relationships between the elemental composition, grain size, and mechanical properties such as the tensile strength. With the inclusion of hardness, the model’s predictive accuracy was markedly enhanced, underscoring the value of incorporating comprehensive mechanical properties into the predictive framework. This advancement in feature selection methodology was validated by improved accuracy metrics, attesting to the efficacy of our approach.

The implications of this study are substantial, offering meaningful contributions to the predictive modeling and design of aluminum alloy tensile strength. This research enriches our understanding of the intricate relationship between the alloy composition, grain size, hardness, and tensile strength, thus paving the way for refined improvement and optimization strategies for aluminum alloys. Furthermore, the methodologies and techniques employed here bear the potential for application across diverse material systems, heralding a new frontier in material performance prediction and material informatics.

## Figures and Tables

**Table 1:** Twenty-two features characterizing aluminum alloys used in analysis.

Input/Output	Abb.	Description	Min	Max	Mean
Inputs	*Al*	Mass fraction of *Al*/%	84.450	100.00	93.626
	*Si*	Mass fraction of *Si*/%	0.000	7.270	1.114
	*Fe*	Mass fraction of *Fe*/%	0.000	0.634	0.209
	*Cu*	Mass fraction of *Cu*/%	0.000	4.420	1.297
	*Mn*	Mass fraction of *Mn*/%	0.000	5.420	0.480
	*Mg*	Mass fraction of *Mg*/%	0.000	6.380	1.628
	*Cr*	Mass fraction of *Cr*/%	0.000	5.600	0.135
	*Zn*	Mass fraction of *Zn*/%	0.000	7.840	1.460
	*Ti*	Mass fraction of *Ti*/%	0.000	1.010	0.073
	*Sc*	Mass fraction of *Sc*/%	0.000	0.100	0.001
	*Er*	Mass fraction of *Er*/%	0.000	0.100	0.001
	*Zr*	Mass fraction of *Zr*/%	0.000	0.200	0.011
	*Ni*	Mass fraction of *Ni*/%	0.000	0.035	0.003
	*B*	Mass fraction of *B*/%	0.000	0.042	0.002
	*Be*	Mass fraction of *Be*/%	0.000	0.001	0.000
	*Li*	Mass fraction of *Li*/%	0.000	2.200	0.033
	*Ce*	Mass fraction of *Ce*/%	0.000	0.100	0.001
	*La*	Mass fraction of *La*/%	0.000	0.210	0.003
	*Sr*	Mass fraction of *Sr*/%	0.000	0.011	0.001
	*Gs*	Grain size/um	0.360	482.000	76.713
	*Hardness*	Hardness/Hv	48.000	302.030	108.838
Output	*Ts*	Tensile strength/MPa	115.000	696.000	349.528

**Table 2:** Performance metrics of ML models for predicting tensile strength in aluminum alloys (alloy composition and grain size).

ML Models	*R^2^*	*RMSE* (MPa)	*MSE* (MPa)	*MAE* (MPa)
KNN	0.393	129.044	16,652.275	99.490
LR	0.550	171.507	29,414.598	85.920
ANN	0.732	85.691	33,282.024	145.833
LightGBM	0.766	80.062	6409.911	63.933
RF	0.858	62.424	3688.174	43.259
XGBoost	0.878	57.721	3331.768	39.267

**Table 3:** Top four high-accuracy machine learning models with screened composition and grain size as input.

ML Models	*R^2^*	*RMSE* (MPa)	*MAE* (MPa)	*MSE* (MPa)
LR	0.501	117.008	93.539	13,690.907
XGBoost	0.884	56.452	40.034	3186.835
RF	0.891	54.689	37.897	2990.903
LightGBM	0.912	49.227	37.675	2423.319

**Table 4:** Four best-performing machine learning models based on accuracy (using screened composition, grain size, and hardness).

ML Models	*R^2^*	*RMSE* (MPa)	*MAE* (MPa)	*MSE* (MPa)
RF	0.854	54.447	40.573	2964.502
LR	0.904	44.217	35.324	1955.187
GBDT	0.907	43.470	31.785	1889.660
XGBoost	0.914	41.740	29.910	1742.203

## Data Availability

All the data are available within the manuscript.
